# A p53-like transcription factor similar to Ndt80 controls the response to nutrient stress in the filamentous fungus, *Aspergillus nidulans*

**DOI:** 10.12688/f1000research.2-72.v1

**Published:** 2013-03-04

**Authors:** Margaret E Katz, Kathryn Braunberger, Gauncai Yi, Sarah Cooper, Heather M Nonhebel, Cedric Gondro

**Affiliations:** 1 Department of Molecular and Cellular Biology, University of New England, Armidale, NSW 2351, Australia; 2 Current address: Nanjing Hospital for Women & Children's Health, Nanjing Medical University, Nanjing City, 210004, China; 3 The Centre for Genetic Analysis and Applications, University of New England, Armidale, NSW 2351, Australia

## Abstract

The *Aspergillus nidulans xprG* gene encodes a putative transcriptional activator that is a member of the Ndt80 family in the p53-like superfamily of proteins. Previous studies have shown that XprG controls the production of extracellular proteases in response to starvation. We undertook transcriptional profiling to investigate whether XprG has a wider role as a global regulator of the carbon nutrient stress response. Our microarray data showed that the expression of a large number of genes, including genes involved in secondary metabolism, development, high-affinity glucose uptake and autolysis, were altered in an *xprG* *Δ* null mutant. Many of these genes are known to be regulated in response to carbon starvation. We confirmed that sterigmatocystin and penicillin production is reduced in *xprG ^-^* mutants. The loss of fungal mass and secretion of pigments that accompanies fungal autolysis in response to nutrient depletion was accelerated in an *xprG1* gain-of-function mutant and decreased or absent in an *xprG ^-^* mutant. The results support the hypothesis that XprG plays a major role in the response to carbon limitation and that nutrient sensing may represent one of the ancestral roles for the p53-like superfamily. Disruption of the AN6015 gene, which encodes a second Ndt80-like protein, showed that it is required for sexual reproduction in *A. nidulans*.

## Introduction

XprG and two non-catalytic hexokinase-like proteins (HxkC and HxkD) were first identified as regulators of extracellular protease production in *Aspergillus nidulans* through genetic analysis ^1– 3^. In *A. nidulans*, extracellular proteases are produced in response to carbon, nitrogen or sulfur starvation ^4^. Genetic evidence indicates that XprG activates expression of extracellular protease genes in response to nutrient stress and that HxkC and HxkD are negative regulators of XprG ^1– 3, 5, 6^. The *hxkCΔ1* and *hxkDΔ3* null mutations and the *xprG1* gain-of-function mutation increase production of extracellular proteases ^1– 3, 5^. In contrast, loss-of-function mutations in *xprG* abolish carbon-starvation-induced production of extracellular proteases and are epistatic to the *hxkCΔ1* and *hxkDΔ3* null mutations ^3, 6, 7^. The production of an acid phosphatase in response to phosphate limitation and of extracellular proteases in response to nitrogen- and sulfur-starvation is also reduced in *xprG ^-^* mutants ^7^. Thus, there is evidence that XprG could be involved in a general response to starvation.

XprG is similar to VIB-1 of *Neurospora crassa*, and both are members of the Ndt80 family of p53-like, Ig-fold transcriptional activators (Pfam PF05224) ^7^. VIB-1 is required for expression of genes involved in heterokaryon incompatibility, a type of programmed cell death (PCD) ^8^. XprG is also similar to the *Saccharomyces cerevisiae* meiosis-specific transcriptional activator, Ndt80 ^9^. Ndt80 activates the transcription of more than 150 genes during the middle phase of meiosis and is required for progression through meiosis ^10^. It has recently been shown that Ndt80 is also involved in resetting lifespan during meiosis and that transient expression of *NDT80* extends the lifespan of aging yeast cells ^11^.

HxkC and HxkD are similar in sequence to catalytic hexokinases but lack some of the conserved residues found in the sugar-binding and ATP-binding domains ^1^. In addition, both possess an extra stretch of amino acids within the adenosine-binding domain. Several plant hexokinase-like proteins that lack catalytic activity also possess an insertion in this same position ^12, 13^. The *hxkC ^-^* and *hxkD ^-^* mutants have similar phenotypic effects on extracellular protease production but the proteins encoded by these genes are located in different subcellular compartments ^1^. HxkD is a nuclear protein and HxkC is the first fungal hexokinase shown to be associated with mitochondria. Binding of hexokinase to mitochondria blocks apoptosis in human cells and PCD in plants ^14– 16^.

As meiosis in *S. cerevisiae* requires nutrient deprivation and genes expressed during heterokaryon incompatibility are also expressed in response to starvation, we have suggested that nutrient sensing may be a feature of all Ndt80 family members ^7^. Previous studies have shown that XprG regulates production of extracellular proteases and an acid phosphatase in response to starvation ^2, 3, 5– 7^. In this report, we show that XprG has a wider role as a global regulator of the carbon nutrient stress response and is involved in triggering autolysis, a form of fungal programmed cell death induced by starvation.

## Materials and methods

### *Aspergillus* media, growth conditions, and genetic techniques

*A. nidulans* was cultured at 37°C in *Aspergillus* complete or minimal medium ^17^ except that glucose was omitted from media that contained other carbon sources. For media that contained 1% skim milk as a carbon source, sodium deoxycholate (0.08%) was used to induce compact colony formation. For RNA extraction, mycelia were grown for 24 h in minimal medium containing glucose and then transferred to minimal medium containing glucose or no carbon source for 16 h. To monitor autolysis, six flasks containing 50 mL of minimal medium, 10 mM ammonium tartrate and vitamin supplements were each inoculated with 3×10 ^8^ conidia and placed on an orbital shaker. Flasks were removed at 24 or 48 h intervals, the submerged mycelia harvested using Miracloth (Calbiochem/Merck) and samples of filtered culture medium collected. To observe conidiophore development on solid medium, strains were inoculated into 1 cm ^2^ blocks of complete medium on microscope slides as described by Larone ^18^. The techniques used for genetic analysis of *A. nidulans* have been described ^19^. The *Aspergillus* strains used in this study are listed in Table 1.

**Table 1.** List of *Aspergillus nidulans* strains used in this study.

Strain	Genotype ^a^	Source
MH2	*biA1; niiA4*	M.J. Hynes
MH97	*pabaA1 yA1 acuE215*	M.J. Hynes
MK85	*biA1; xprG1; niiA4*	Katz *et al.* [ 2]
MK86	*suA-adE20 yA1 adE20; xprG1; niiA4 riboB2*	Katz *et al.* [ 2]
MK186	*yA1 acuE215; prnΔ309 hxkD1 xprG2; niiA4 riboB2*	Katz *et al.* [ 3]
MK198	*pabaA1; prnΔ309 xprG2; niiA4*	Katz *et al.* [ 3]
MK320	*pabaA1 yA2; argB2; hxkDΔ3 (hxkD::argB)*	Bernardo *et al.* [ 1]
MK388	*pabaA1 yA2; hxkCΔ1 (hxkC::argB); argB2*	Bernardo *et al.* [ 1]
MK408	*pabaA1 yA2; hxkCΔ1(hxkC::argB); argB2 amdS::lacZ; xprG2*	Bernardo *et al.* [ 1]
MK413	*pabaA1 yA2; argB2; xprGΔ2(xprG::argB)*	Katz *et al.* [ 7]
MK414	*pabaA1 yA2; argB2; xprGΔ2(xprG::argB)*	Katz *et al.* [ 7]
MK422	*biA1; xprGΔ1(xprG::argB)*	Katz *et al.* [ 5]
MK481	*ndtAΔ (ndtA::A. fumigatus pyroA); pyroA4 nkuA::argB; riboB2*	This study
MK505	*ndtAΔ (ndtA::A. fumigatus pyroA); pyroA4 nkuA::argB; prnΔ309 xprG2; niiA4*	This study
MK531	*ndtAΔ (ndtA::A. fumigatus pyroA) yA2; hxkCΔ1 (hxkC::argB);argB2; pyroA4*	This study
MK532	*ndtAΔ (ndtA::A. fumigatus pyroA) pabaA1 yA2; argB2; pyroA4 nkuA::argB; hxkDΔ3 (hxkD::argB)*	This study
MK563	*biA1; xprG1; veA ^+^*	This study
MK565	*pabaA1; xprG2; veA ^+^*	This study
MK592	*biA1; fluG701*	This study
MK593	*pabaA1 yA2; fluG701*	This study
MK594	*biA1; fluG701 xprG1*	This study
MK595	*pabaA1 yA2; fluG701; xprG1*	This study
WIM-126	*pabaA1 yA2; veA ^+^*	Butnick *et al.* [ 68]

^a^The gene symbols are described in the Aspergillus Genome Database.

### RNA extraction and qRT-PCR

Total RNA was prepared using a procedure developed by Reinert *et al.* ^20^. mRNA was prepared from total RNA using the PolyATtract® mRNA Isolation System IV as described by the manufacturer (Promega Corp.). DNA was removed from total RNA or polyA+ RNA with the Ambion Turbo DNA-free Kit™ (Applied Biosystems) prior to quantification with a NanoDrop® spectrophotometer. The primers ( Supplementary Table 1) used in qRT-PCR experiments were designed using the Primer3 program ( http://frodo.wi.mit.edu/primer3/). Each primer pair was first tested with serial dilutions of MH2 RNA to determine the linear range of the qRT-PCR assays using SuperScript III Platinum SYBR Green One-Step qRT-PCR Kits (Invitrogen). The experiments were performed using a Corbett CAS1200 liquid handling robot and Corbett Rotor-Gene 3000 real-time thermal cycler (QIAGEN). In the assays to determine relative transcript levels, 1 ng of total RNA was added to each reaction. Each reaction was performed in duplicate or triplicate and the *actA* control reactions were included in each run.

### cDNA labeling, microarray hybridization and scanning

cDNAs labeled with Alexa Fluor® 555 and Alexa Fluor® 647 were prepared from mRNA using the SuperScript™ Plus Indirect cDNA Labeling System according to the instructions of the manufacturer (Invitrogen). *A. nidulans* DNA microarrays, supplied by the Pathogen Functional Genomics Resource Center (PFGRC) at The Institute for Genomic Research (TIGR) were hybridized with the labeled cDNAs using the TIGR protocol ^21^. The *A. nidulans* microarrays consisted of 11,481 unique 70-mer oligonucleotides spotted in duplicate on the array plus an additional 1,000 control probes from *Arabidopsis thaliana* and 1,430 empty features (negative controls). The hybridized slides were scanned immediately in an Axon 4200AL scanner (Molecular Devices). The intensity values for the two channels for each spot were acquired by automatic photomultiplier tube gains to obtain the highest intensity with 0.05% saturated pixels. The resulting images were analyzed by measuring the fluorescence of all features on the slides using GenePix Pro 6.1 software (Molecular Devices). The median fluorescence intensity of these pixels within each feature was taken as the intensity value for the feature.

### Microarray data analysis

The NCBI Gene Expression Omnibus (GEO) accession number for the microarray data reported in this paper is GSE36235 and the data are available at http://www.ncbi.nlm.nih.gov/geo/. Also available for download from this GEO accession is a *Supplementary Analysis File* containing all pre-processing analyses, annotated lists of differentially expressed genes with links to NCBI as well as gene ontology, pathway analyses and other relevant images and diagrams ( http://www.ncbi.nlm.nih.gov/geo/query/acc.cgi?acc=GSE36235&submit.x=15&submit.y=14).

Quality control measures, pre-processing and analyses were performed using the statistical computing language R ^22^ and Bioconductor ^23^. All microarray images and quality control measurements were within recommended limits ^24^. The quality of the arrays was assessed through standard quality control measures: pseudo-images of the arrays (to detect spatial effects), MA (M is the intensity ratio and A is the average intensity) scatter plots of the arrays versus a pseudo-median reference chip, and other summary statistics including histogram and boxplots of raw log intensities, signal-to-noise ratios on both channels, boxplots of plates and print tips, boxplots of normalized log ratios, among others. Transcription intensities in adjusted log2 were estimated after normalization within arrays using maximum likelihood ^25^ followed by between array variance stabilization ^26^. Briefly, the data were adjusted by an affine transformation and then all slides were log2 transformed to stabilize the variance. Prior to testing for differential expression, the data were filtered to remove control (n=1,000 from *Arabidopsis thaliana*) and empty spots (n=1,430) and spots flagged as bad in over 90% of the slides (n=4,754), thus leaving 9,104 unique features to be tested.

Differential expression was tested on a gene by gene basis using a moderated t-test with intensities adjusted using an Empirical Bayes approach ^27^. A covariance structure to account for the duplicate probes and within array variability was also fitted to the model. Features were considered significantly differentially expressed for a false discovery rate adjusted p-value of 0.05 using the Benjamini-Hochberg correction ^28^.

### Annotation and functional analysis of differentially expressed probes

The annotation of the array features was derived from the AspGD – *Aspergillus* Genome Database ^29^ and identifiers were annotated to gene ontology terms and pathway information for testing gene set enrichment in GO and KEGG (Kyoto Encyclopedia of Gene and Genomes). In subsequent text the term probe is replaced by gene. The differentially expressed genes were analyzed in the context of their Gene Ontology (GO) ^30^ and involvement in KEGG biological pathways ^31, 32^.

Functional profiles for the differentially expressed genes were derived for each of the GO categories: cellular component, molecular function and biological process. Differentially expressed genes were mapped from their Entrez identifier to their most specific GO term and these were used to span the tree structure and test for gene enriched terms. Profiles for each category were also constructed for the differentially expressed genes for different tree depths ( Supplementary Analysis File). To avoid over-inflated p-values, the background for both GO and KEGG pathway analyses consisted exclusively of the array probes used in the analyses after the removal of control probes, unexpressed probes and unannotated probes. Gene ontologies and KEGG pathways reported in this manuscript include those with a significance value of p < 0.05.

### Extraction and detection of sterigmatocystin

For sterigmatocystin assays, flasks containing 50 mL of *Aspergillus* minimal medium were inoculated with 3 x 10 ^8^ conidia scraped from cultures grown on complete medium containing 2.2% agar. After 24 h, the growth medium was collected and the mycelia were transferred to carbon-free medium for 24 h. Sterigmatocystin was extracted from 10 mL aliquots of filtered growth medium using the method described by Keller *et al.* ^33^ with the following modifications. An equal volume of chloroform was added to each sample, mixed vigorously and agitated on a shaking platform for 15 min. After centrifugation at 1600 x *g* for 5 min, the aqueous phase was transferred to a fresh tube and the chloroform extraction was repeated. The chloroform from the first and second extractions was pooled, dried in a rotary evaporator and the residue resuspended in 50 µL chloroform. A 5 µL sample of each extract was applied to aluminum-backed, silica thin layer chromatography sheets (Merck) and separated using a mixture of benzene and glacial acetic acid (95:5). After drying, the plate was sprayed with 15% AlCl _3_ dissolved in 95% ethanol, baked at 65°C for 15 min and photographed under 365 nm UV illumination. Sterigmatocystin (Sigma) was used as a standard.

Sterigmatocystin was also extracted from three 16 mm plugs taken from conidiating colonies grown on solid minimal medium using the method described by Keller *et al.* ^33^ with the following modifications. Chloroform (1 mL) was added to the agar plugs and mixed vigorously. After centrifugation at 1000 x *g* for 5 min, the chloroform containing the extracted sterigmatocystin was transferred to a fresh tube, washed twice with 0.5 mL Milli-Q water (QPAK 2 purification pack, Millipore) and then evaporated. The residue was resuspended in 0.1 mL chloroform.

### Penicillin bioassays

Penicillin levels in filtered penicillin production broth containing 3% lactose or 3% glucose were assayed as described by Espeso and Peñalva ^34^. 5 mL aliquots of filter-sterilized culture medium were lyophilised and resuspended in 300 µL of 10 mM sodium phosphate buffer pH 6.8. The volume (35–50 µL) corresponding to the penicillin produced by 9.3 mg mycelium (dry weight) was applied to 6 mm wells in Luria Broth plates seeded with *Micrococcus luteus* (UNE014). Penicillin G (Sigma) dissolved in 10 mM sodium phosphate buffer pH 6.8 was applied as a control. The filtrates were left to diffuse for 18 h at 4°C and then incubated at 30°C for 32 h. For samples treated with penicillinase (Sigma Aldrich), 1 µL containing 1 U of enzyme in 100 mM Tris-HCl pH7 with 0.1% BSA was added and the samples were incubated at 25°C for 15 min before they were applied to the plates. The samples that were not treated with penicillinase were treated in an identical manner except that the 1 µL of 100 mM Tris-HCl pH7 0.1% BSA did not contain any enzyme.

### Glucose uptake assays

The uptake of D-[U- ^14^C] glucose (10.6 GBq/mmol, Amersham) was measured in germinating conidia as described previously ^35^. Conidia were germinated in minimal medium containing 1% glucose, 0.1% yeast extract, 10 mM ammonium tartrate and vitamins and then washed five times with carbon-free minimal medium containing 10 mM NH _4_Cl and vitamins. Glucose uptake was measured in aliquots of 2.5 x 10 ^7^ germinating conidia 5, 30, 60 and 90 s after transfer to media containing 0.025, 0.125, 0.5 or 2 mM glucose.

### Disruption of AN6015

The AN6015 gene ( *ndtA*) was disrupted in an *nkuAΔ* strain (MH11036) so as to increase the frequency of gene targeting events ^36^. The entire predicted coding region of AN6015 (nucleotides 21661–23381, contig 103; *Aspergillus* Comparative Database) was replaced with the *Aspergillus fumigatus* *pyroA* gene using a similar strategy to the one described in Nayak *et al.* ^36^. Gene disruption was confirmed by PCR and Southern blot analysis. Double mutants with lesions in AN6015 ( *ndtA*) and *hxkC*, *hxkD* or *xprG* were generated in crosses and the presence of *ndtA::A. fumigatus pyroA* was confirmed by PCR using primers MK261 (5´-AACGGTTACCTCCCAATTGC-3´) complementary to sequences upstream of the *A. nidulans ndtA* coding region and MK323 (5´-GATGGTCTCGAACTGACCTT-3´) complementary to the *A. fumigatus pyroA* gene.

## Results

### Transcriptional profiling

**Figure 1.**
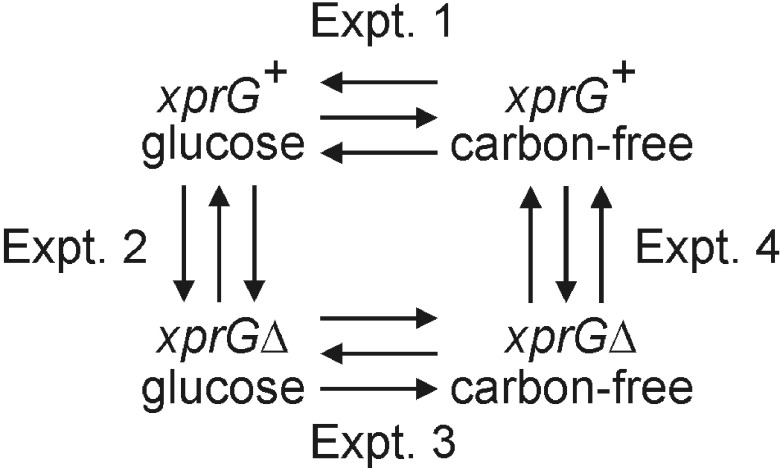
Design of the microarray experiments. The arrowheads point to the samples labeled with Alexa Fluor® 555. Each experiment consisted of three biological replicates, indicated by arrows, and included a dye swap. The full genotypes of the *xprG ^+^* (MH2) and *xprGΔ* (MK422) strains are given in Table 1.

**Figure 2.**
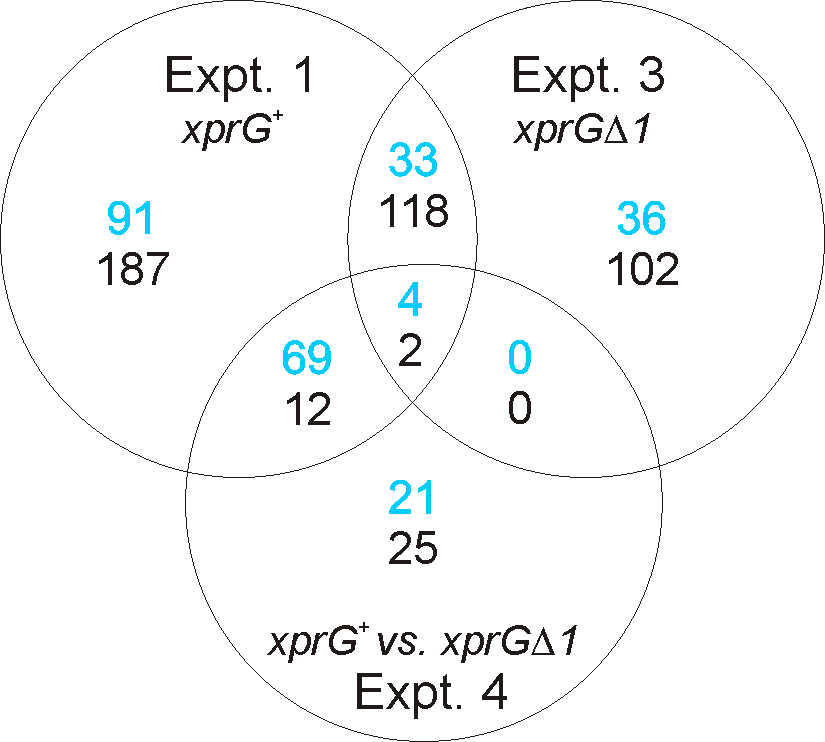
Venn diagram showing the number of probes hybridising to differentially expressed transcripts in different *Aspergillus nidulans* genotypes. In Experiments 1 and 3, the number of transcripts up-regulated during carbon starvation is shown in blue and the number down-regulated is shown in black. In Experiment 4, the number of transcripts that are down-regulated in the *xprGΔ1* mutant is shown in blue and number up-regulated is shown in black.

*A. nidulans* microarrays provided by the Pathogen Functional Genomics Resource Center (PFGRC) were used to compare transcript levels in an *xprG ^+^* strain and an *xprGΔ* null strain after transfer to medium containing glucose as a carbon source or medium lacking a carbon source (carbon starvation) for 16 h. These four experiments ( Figure 1) were designed to detect differences in transcript levels between the two strains (Experiments 2 and 4) and changes in transcript levels in each strain due to the different nutrient conditions (Experiments 1 and 3). The NCBI Gene Expression Omnibus (GEO) accession number for the microarray data reported in this paper is GSE36235 and is available at http://www.ncbi.nlm.nih.gov/geo/. A total of 516 probes that hybridized to differentially expressed transcripts were detected in Experiment 1, which examined the effect of carbon starvation in an *xprG ^+^* strain. One hundred and ninety seven were up-regulated and 319 were down-regulated during carbon starvation ( Figure 2). The top five biological processes identified in the Gene Ontology analysis of Experiment 1 were sterigmatocystin biosynthesis, ergosterol biosynthesis, conidial spore wall assembly, the purine salvage pathway and autolysis. In the *xprGΔ1* mutant, the number of transcripts that showed a significant change in response to carbon starvation was lower ( Figure 2). All of the 73 up-regulated and 222 down-regulated transcripts in Experiment 3 showed similar responses (in direction) to carbon starvation in Experiment 1.

In Experiment 4, which examined the effect of the *xprGΔ1* mutation on *A. nidulans’* response to carbon starvation, 133 probes hybridized to transcripts that were either up- or down-regulated ( Figure 2). Ninety four probes hybridized to transcripts that were down-regulated in the *xprGΔ1* mutant and 39 genes were up-regulated. Fifteen of the down-regulated transcripts, including four of the top five, belonged to the sterigmatocystin gene cluster ( Table 2). The pathway for the synthesis of sterigmatocystin, a carcinogen closely related to aflatoxin, is encoded by a cluster of 25 co-regulated genes ^37^. Transcripts from an additional four genes from the cluster ( *aflR*, *stcA*, *stcO*, and *stcS*) had lower levels in the *xprGΔ1* mutant with p-values of less than 0.05 prior to applying the Benjamini-Hochberg correction ^28^. The *tdiB* gene, which is down-regulated in the *xprGΔ1* mutant, belongs to another secondary metabolism gene cluster, *tdiA-E*, that controls the biosynthesis of the anti-tumor compound terrequinone A ^38, 39^. A second gene in the cluster, *tdiA*, was down-regulated in the *xprGΔ1* mutant with a p-value of 0.002 prior to adjustment and 0.073 after application of the Benjamini-Hochberg correction. It is interesting that disruption of the *laeA* gene, which encodes another regulator of the *tdi* gene cluster, produced similar effects on the members of the cluster; the reduction in *tdiB* transcript levels was greater than that of *tdiA* and the levels of the *tdiC*, *D* and *E* transcripts were affected to an even lesser extent in the *laeAΔ* mutant ^39^.

**Table 2.** Genes that show altered expression in the *xprGΔ1* mutant during carbon starvation.

Biological process	Genes ^a^	Effect of *xprGΔ1* ^b^
Secondary metabolism	Sterigmatocystin gene cluster: *stcB* (-3.7), *stcE* (-6.2), *stcI* (-5.4), *stcL* (-5.7), *stcN* (-4.5), *stcQ* (-3.3), *stcT* (-4.4), *stcU* (-9.0), *stcV* (-4.8), *stcW* (-4.4), AN7809 (-4.5), AN7817 (-4.8), AN7819 (-3.8), AN11017 (-5.4), AN11021 (-4.1) *tdiB* (-4.8)	down down
Conidiophore development	*brlA* (-4.9), *ivoC* (-4.4), hydrophobins: *rodA* (-3.2), AN0940 (-4.3), AN1873 (-5.1), AN6401 (-3.3)	down
Sexual reproduction	*ppgA* (5.1), *preA* (5.2), *veA* (2.8)	up
Extracellular protease production	*hxkC* (-6.1), *pepJ* (-5.3), *prtA* (-3.2)	down
Autolysis/apoptosis	*chiB* (-3.3), *nagA* (-4.6)	down
Sugar transport (high affinity)	*mstA* (-4.0)	down
Other	*aciA* (2.9), *gabA* (3.2), *gltA* (3.7), CYP680A1 (3.4) *agdB* (-3.5), *atrD* (-3.7), *H4.1* (-2.2)	up down

^a^The genes are described in the Aspergillus Genome Database. Only named genes (and genes with a similar function to the named genes) are listed. The fold change (log2 scale) is given in parentheses, with a negative value indicating that the gene is down-regulated in the *xprGΔ1* mutant during carbon starvation. The full data set for differentially expressed genes is available through NCBI Gene Expression Omnibus (GEO) accession number GSE 36235, http://www.ncbi.nlm.nih.gov/geo/query/acc.cgi?acc=GSE36235).

^b^The effect of the *xprGΔ1* mutation on transcript levels during carbon starvation was determined in microarray experiments.

Other genes with documented functions that showed differential expression in response to carbon starvation in the *xprGΔ1* mutant include two genes encoding extracellular proteases ( *prtA* and *pepJ*) which are known to be expressed during starvation ^5, 40, 41^. The expression of *prtA* in response to carbon or nitrogen starvation has been shown to be XprG-dependent ^6^. HxkC is involved in the regulation of extracellular protease production. Disruption of the *hxkC* gene, which is down-regulated in the *xprGΔ1* mutant, increases extracellular protease production ^1^.

The microarray data indicated that a key regulator of conidiophore development *brlA* ^42^ was down-regulated in the *xprGΔ1* mutant, while the *veA* gene, which activates sexual development ^43^ was up-regulated. Genes encoding a putative sex pheromone ( *ppgA*) and pheromone receptor ( *preA*) were also expressed at higher levels in the *xprGΔ1* mutant. Carbon starvation is known to induce transcription of the *brlA* gene ^44^.

Autolysis is a process of hyphal fragmentation and digestion that occurs in stationary cultures of *A. nidulans* after carbon source depletion ^45^. Though autolysis and apoptotic cell death occur concurrently during carbon starvation, genetic evidence indicates that the two processes are regulated independently ^46^. The chitinase encoded by the *chiB* gene plays an important role in autolysis ^47^ while *nagA* is involved in apoptotic cell death ^48^. Both *chiB* and *nagA*, which were up-regulated in response to carbon starvation in the *xprG ^+^* strain in Experiment 1, are down-regulated in the *xprGΔ1* mutant.

In contrast to Experiment 4, only two probes on the array showed significantly different intensities when hybridized with cDNA prepared from *xprG* ^+^ and *xprGΔ1* strains grown in medium containing glucose in Experiment 2. This confirms that the role of XprG is mainly confined to the starvation response. Only one of the two probes identified in Experiment 2 is annotated as a gene, *hpdA*, which encodes a putative 4-hydroxyphenylpyruvate dioxygenase with a predicted role in pyomelanin production. In *Aspergillus fumigatus*, disruption of the *hpdA* homolog ( *hppD*) abolished pyomelanin pigment production and no pigment was detected in mycelia or culture medium of the mutant when it was grown in liquid medium ^49^.

### qRT-PCR validation

**Supplementary Figure 1.**
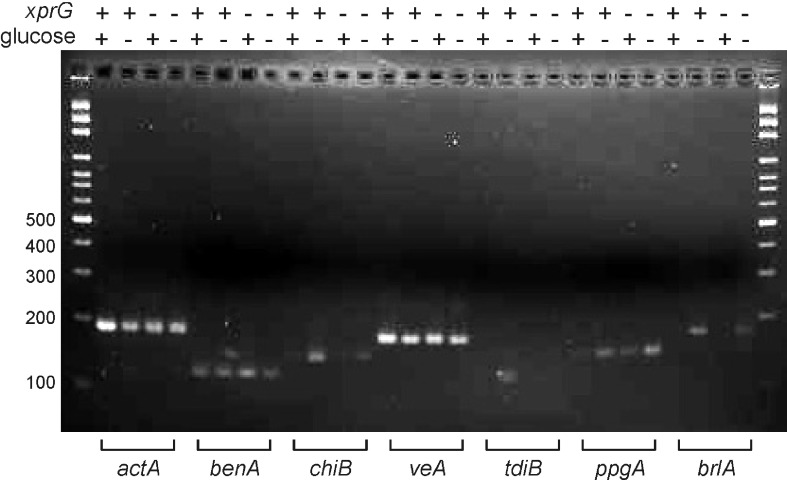
Agarose gel electrophoresis of qRT-PCR products. The template was total RNA extracted from an *xprG ^+^* (+) and *xprGΔ1* (-) strains transferred to glucose (+) or carbon-free medium (-) for 16 h. A 100 bp ladder (Axygen) was used as a standard in the first and last lanes of the 2% agarose gel. The full genotypes of the *xprG ^+^* strain (MH2) and the *xprGΔ1* strain (MK422) are given in Table 1.

Three genes that were down-regulated ( *brlA*, *chiB*, *tdiB*) and two that were up-regulated ( *ppgA*, *veA*) in the *xprGΔ1* mutant (Experiment 4) were analyzed in qRT-PCR experiments using new preparations of RNA ( Table 3), and by agarose gel electrophoresis of qRT-PCR products ( Supplementary Figure 1). The housekeeping gene encoding actin ( *actA*) was used as a control. The level of the actin transcript was lower in carbon-free medium than in glucose in both strains. In previous studies we have observed, using Northern blot analysis, that the level of the *actA* transcript is reduced (relative to rRNAs) during carbon starvation ^5^. The transcript levels in the three down-regulated genes were all higher in the *xprG ^+^* strain than in *xprGΔ1* mutant during carbon starvation and were higher during carbon starvation than in nutrient-sufficient conditions in a *xprG ^+^* strain as predicted by the microarray results. The qRT-PCR data for the up-regulated *ppgA* gene showed much higher expression in the *xprGΔ1* mutant than the wild-type strain during carbon starvation and higher levels in carbon-free medium than glucose for the *xprGΔ1* mutant, consistent with the results in microarray Experiments 4 and 3, respectively. However, no significant difference in *ppgA* expression was detected in microarray Experiment 1, whereas the qRT-PCR data suggest that *ppgA* transcript levels are higher during carbon starvation in the *xprG ^+^* strain. For the *veA* gene, no differences between the wild-type and mutant strains were detected.

**Table 3.** Results of qRT-PCR validation experiments ^a^.

Gene		Relevant genotype/carbon source			
		*xprG* ^+^/glucose	*xprG* ^+^/carbon-free	*xprGΔ1*/glucose	*xprGΔ1*carbon-free
*actA*	Ct	20.45 ± 0.18	22.15 ± 0.21	20.40 ± 0.20	22.66 ± 0.33
	REL	1	0.35	1.02	0.34
*brlA*	Ct	32.51 ± 0.06	27.28 ± 0.04	34.45 ± 0.86	31.14 ± 0.27
	REL	1	1.69	0.82	1.17
*chiB*	Ct	28.08 ± 0.27	20.37 ± 0.37	27.89 ± 0.26	23.79 ± 0.19
	REL	1	12.60	0.72	3.13
*tdiB*	Ct	30.96 ± 0.07	28.23 ± 0.04	31.98 ± 0.20	31.34 ± 0.78
	REL	1	1.82	0.75	0.86
*ppgA*	Ct	32.71 ± 1.70	29.26 ± 0.24	29.83 ± 0.38	24.51 ± 0.06
	REL	1	4.35	2.68	24.3
*veA*	Ct	24.41 ± 0.08	27.13 ± 0.10	24.32 ± 0.18	25.72 ± 0.22
	REL	1	0.72	1.00	0.83

^a^The average cycle threshold (Ct) values for threshold of 0.03 normalized fluorescence units and standard errors are shown. A lower Ct value indicates higher transcript levels. Relative expression levels (REL), based on the Takeoff point and reaction efficiency, were calculated using the Corbett Rotor-Gene Comparative Quantitation program, using the *xprG* ^+^/glucose reactions for each gene as the calibrator. The relative expression levels do not take into consideration the differences in the *actA* transcript levels.

### Secondary metabolism in xprG mutants

**Figure 3.**
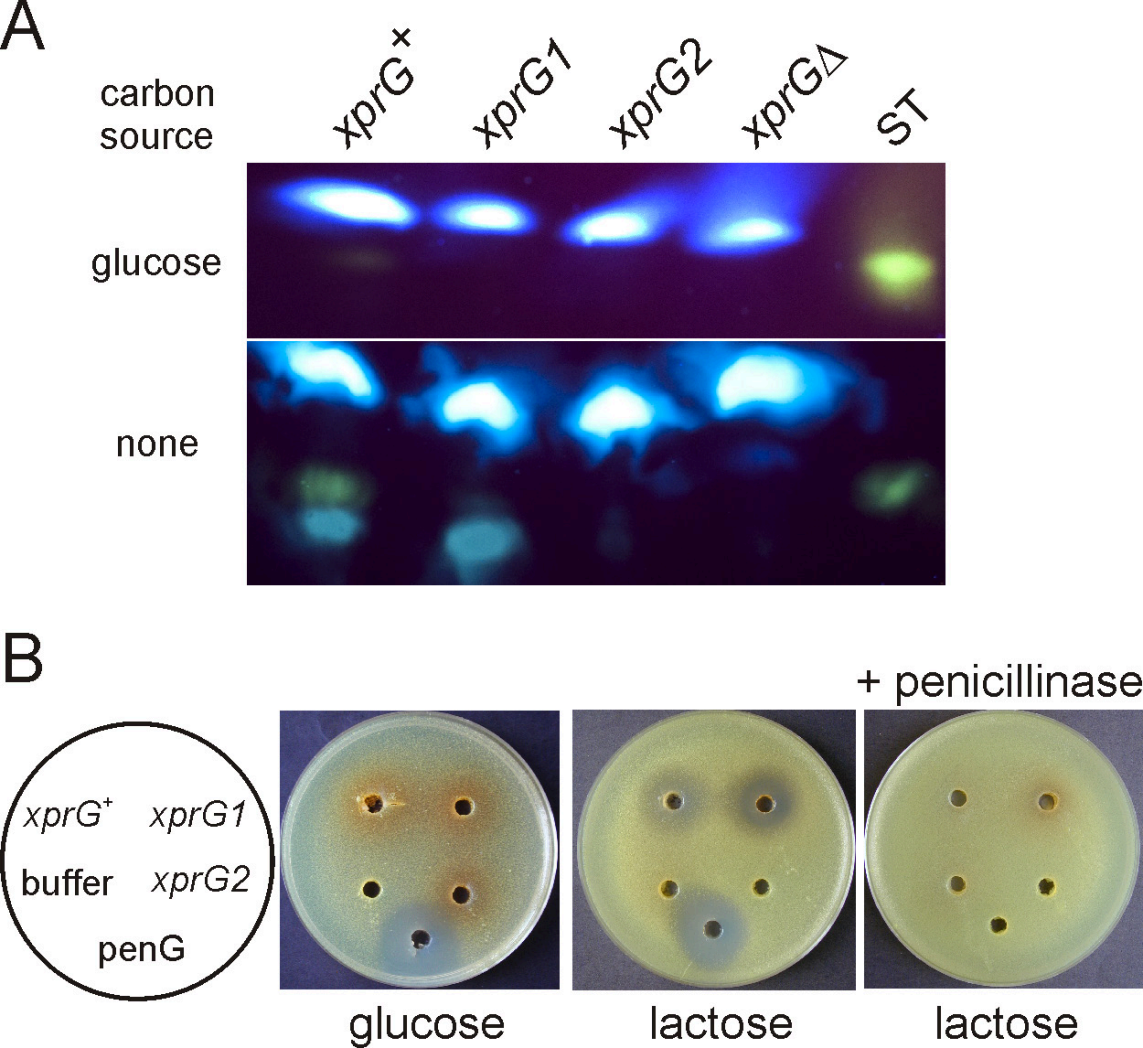
Sterigmatocystin (A) and penicillin (B) production in *xprG* loss-of-function ( *xprG ^-^*) and gain-of-function ( *xprG1*) mutants. **A.** Sterigmatocystin, extracted from the filtered growth medium of an *xprG ^+^* strain (MH2), an *xprG1* strain (MK85) and two *xprG ^-^* strains, MK198 ( *xprG2)* and MK422 ( *xprGΔ1*), was analyzed using thin layer chromatography. Sterigmatocystin fluoresces yellow after treatment with AlCl _3_. Sterigmatocystin (ST) (Sigma) was applied as a standard. The cultures used in the assays were generated by inoculating growth medium with 3 x 10 ^8^ conidia. After transfer to carbon-free medium for 24 h, the dry mycelial weights were 100 mg ( *xprG ^+^*), 71 mg ( *xprG1*), 129 ( *xprG2*) and 152 mg ( *xprGΔ1*). **B.** Penicillin bioassay based on inhibition of bacterial growth. Samples of filtered, concentrated growth medium from strains MH2 ( *xprG ^+^*), MK85 ( *xprG1*), and MK198 ( *xprG2*) was applied to wells in medium seeded with the *Micrococcus luteus*. 400 ng of penicillin G (penG) and 10 mM sodium orthophosphate buffer pH 6.8 (buffer) were used as controls. The *Aspergillus* growth medium contained either 3% glucose or 3% lactose. In the right-hand plate the samples were treated with 1 U of penicillinase (Sigma Aldrich) before they were applied to the wells. The full genotypes of the strains are given in Table 1.

**Supplementary Figure 2.**
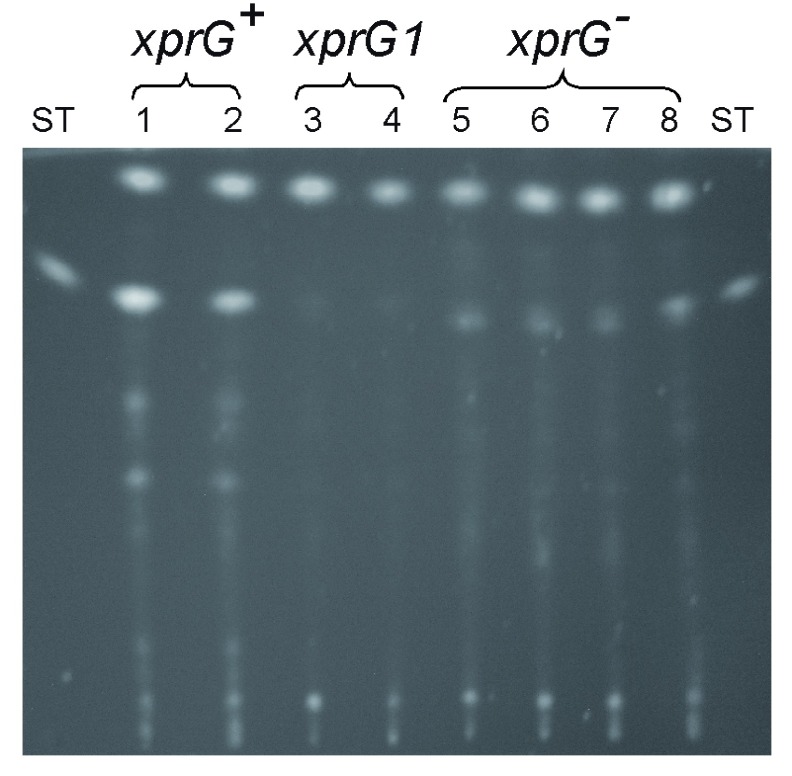
Sterigmatocystin extracted from cultures grown on solid medium. Samples of two *xprG ^+^* strains (lane 1 MH2, Lane 2 MH97), two *xprG1* strains (lane 3 MK85, lane 4 MK86) and four *xprG ^-^* strains (lane 5 MK198, lane 6 MK413, lane 7 MK414, lane 8 MK422) was analyzed using thin layer chromatography with a benzene: glacial acetic acid (95:5 vol/vol) as described in the experimental procedures. A sterigmatocystin (ST) standard (Sigma) was applied as standard).

The results of the microarray experiments suggested that expression of genes in the sterigmatocystin gene cluster was reduced in the *xprGΔ1* mutant. To confirm that sterigmatocystin levels were altered, sterigmatocystin was extracted from the growth medium of strains carrying two different *xprG ^-^* mutations ( *xprG2* and *xprGΔ1*) and a strain carrying the *xprG1* gain-of-function mutation. The *xprG2* loss-of-function mutation is due to the insertion of two base pairs which causes a frameshift mutation in the ninth codon of the *xprG* gene ^7^. The *xprGΔ1* mutation, which lacks codons 248–344, was constructed by gene disruption and has a phenotype that is identical to the *xprG2* mutant ^7^. The *xprG1* mutation is a missense mutation in the putative DNA-binding domain of XprG ^7^. Sterigmatocystin levels were reduced in both the gain- and loss-of-function mutants ( Figure 3A). In the wild-type strain very low levels of sterigmatocystin were detected after 24 h growth in medium containing glucose and much higher levels after transfer, for 24 h, to medium lacking a carbon source. No sterigmatocystin was detected in the *xprG2* or *xprGΔ1* mutants in either growth condition and the level of sterigmatocystin in the *xprG1* gain-of-function mutant was much lower than in the wild-type strain. Production of a blue-green pigment which co-migrates with sterigmatocystin ^33^ was reduced in the *xprG ^-^* mutants but not in the gain-of-function mutant. Sterigmatocystin production was also reduced in the *xprG1*, *xprG2* and *xprGΔ1* cultures grown on solid medium ( Supplementary Figure 2).

Penicillin is also a product of secondary metabolism in *A. nidulans*. Although no significant changes in the expression of penicillin biosynthetic genes were detected in the microarray experiments, this may have been due to the fact that the growth medium was not optimal for penicillin production. Bioassays were used to detect penicillin levels in broth cultures optimised for penicillin production ^34^. The results showed that penicillin levels, as measured by bacterial growth inhibition, were greatly reduced in an *xprG2* loss-of-function mutant and increased in an *xprG1* gain-of-function mutant ( Figure 3B). When glucose was included in the growth medium, no penicillin was detected in the culture medium of any strains ( Figure 3B).

### Effect of xprG mutations on conidiophore development

**Figure 4.**
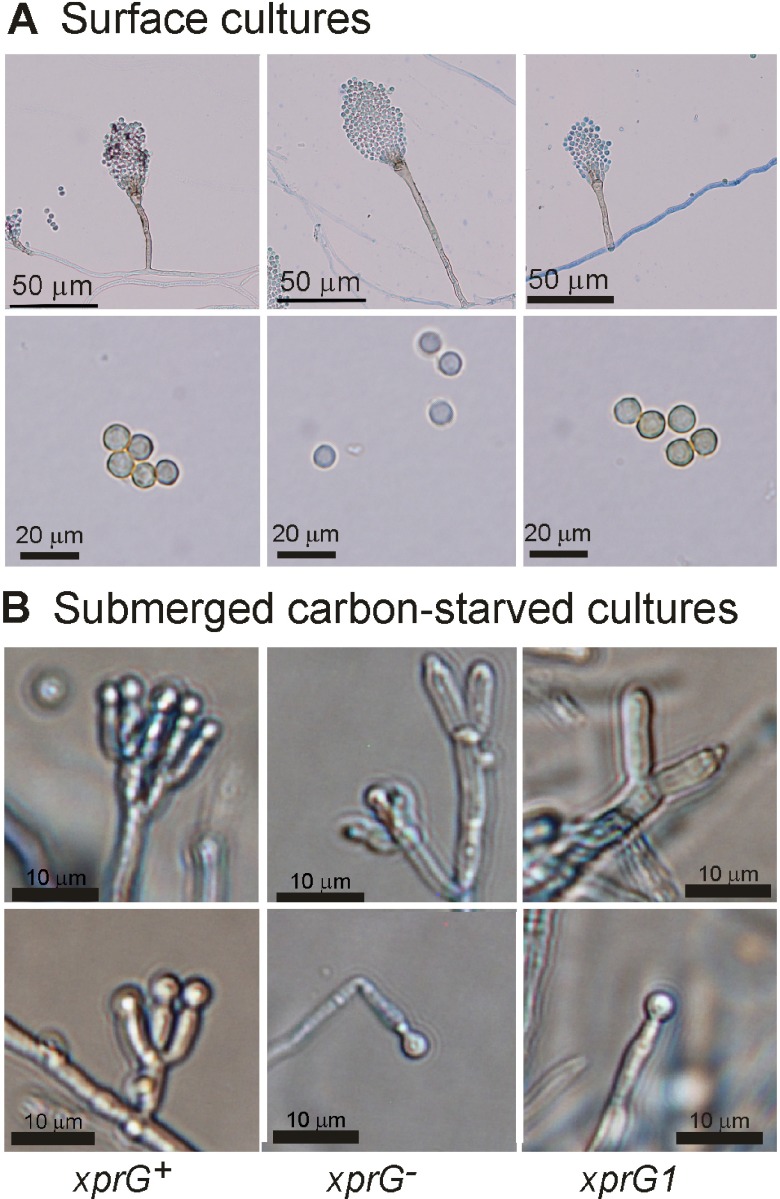
Conidiophore morphology in *xprG ^-^* loss-of-function (middle) and *xprG1* gain-of-function (right) mutants. **A.** Conidiophores of strains MH2, MK198, and MK85 were photographed after 2 days growth at 37°C on solid complete medium on microscope slides followed by treatment with diluted Lactophenol Cotton Blue stain. For the lower set of pictures, conidia were scraped from MH2, MK422 and MK85 colonies on complete medium. Scale bars: 50 µm (upper row), 20 µm (lower row). **B.** Conidiophores of strains MH2, MK422 and MK85 after transfer to carbon-free liquid medium for 24 h. Scale bars: 10 µm. The full genotypes of the *xprG ^+^* (MH2), *xprG2* (MK198), *xprGΔ1* (MK422) and *xprG1* (MK85) strains are given in Table 1.

BrlA is a DNA-binding protein that is required for conidiophore development ^42, 50^. The microarray and qRT-PCR data showed that expression of *brlA* is induced during carbon-starvation but is at lower levels in the *xprGΔ1* mutant. The RNA used in the microarray and qRT-PCR experiments was extracted from mycelia grown in submerged cultures. While conidiation does not normally occur under these conditions, transfer to medium lacking a carbon source does induce conidiation in submerged cultures ^44^. All *xprG ^-^* mutants produce conidia though they are abnormally pale in color ^7^ ( Figure 4A). The conidophore structure of *xprG* mutants was examined and appeared to be normal ( Figure 4A, Table 4). The conidiophore stalk length was highly variable in all strains but the difference between the *xprG ^+^* and *xprG2* is marginally significant (p = 0.05). Asexual spore production was also highly variable in the gain- and loss-of-function mutants ( Table 4). Both *xprG1* and *xprG* ^-^ mutants were slightly slower to initiate conidiophore development.

**Table 4.** Conidiophore development in *xprG* mutants.

Phenotype	Relevant genotype ^a^		
	*xprG* ^+^	*xprG* ^-^	*xprG1*
Conidiophore morphology in surface cultures	normal	normal	normal
Mean conidiophore stalk length ^b^	57.4 ± 19.5 µm	62.5 ± 19.6 µm*	55.7 ± 18.5 µm
Mean no. of conidia per mm ^2^ ^c^	1.23 ± 0.07	1.06 ± 0.33	0.76 ± 0.49
Conidial pigmentation	present	reduced	present
Conidiophore development in submerged cultures ^d^	yes	yes	yes

^a^The full genotypes are given in Table 1. Strains MH2 ( *xprG* ^+^) and MK85 ( *xprG1*) were used for all analyses. Strain MK422 was used for all *xprG* ^-^ analyses except for mean conidiophore stalk length, which used MK198 ( *xprG* ^-^). Conidiophore morphology in surface cultures was examined in both MK198 and MK422.

^b^Conidiophores were photographed at 400 x magnification after growth at 37°C on microscope slides. Measurements were carried out using the ImageJ program ( http://rsbweb.nih.gov/ij/). The mean length (± SD) for over 100 conidiophores are given. The difference between the *xprG* ^-^ and *xprG* ^+^ strains was marginally significant (unpaired t-test, p=0.05)

^c^The number of asexual spores (conidia) per mm ^2^ was determined by removing three plugs from colonies on complete medium containing 2.2% agar. The conidia from each plug were suspended in a solution of 0.01% TWEEN80 and counted in a haemocytometer. The number per mm ^2^ (± SD) is the mean from four experiments which used different batches of media. No significant differences were found using an unpaired t-test.

^d^Conidiophore development was monitored after transfer to carbon-free medium.

Expression of the *ivoC* gene was lower in the *xprGΔ1* mutant. IvoC encodes a putative cytochrome P450 that is required for conidiophore pigmentation (A.J. Clutterbuck, personal communication). The *ivoB* gene also showed lower expression in the *xprGΔ1* mutant with an unadjusted p-value of 0.002. Mutants lacking a functional copy of *ivoA*, *B* or *C* have ivory-coloured conidiophores ^42^. Microscopic examination showed that the conidiophore stalks of *xprG2* mutants display normal pigmentation ( Figure 4A).

Initiation of conidiophore development occurs irrespective of nutrient limitation in *A. nidulans* cultures exposed to air ^51^ and can be induced in submerged cultures by carbon starvation ^44^. We found that conidophore development occurred in carbon-starved submerged cultures of both the *xprGΔ1* loss- and *xprG1* gain-of-function mutants, though the number of metulae appeared to be reduced ( Figure 4B). Thus, XprG is not essential for triggering conidiophore development in response to carbon starvation.

**Figure 5.**
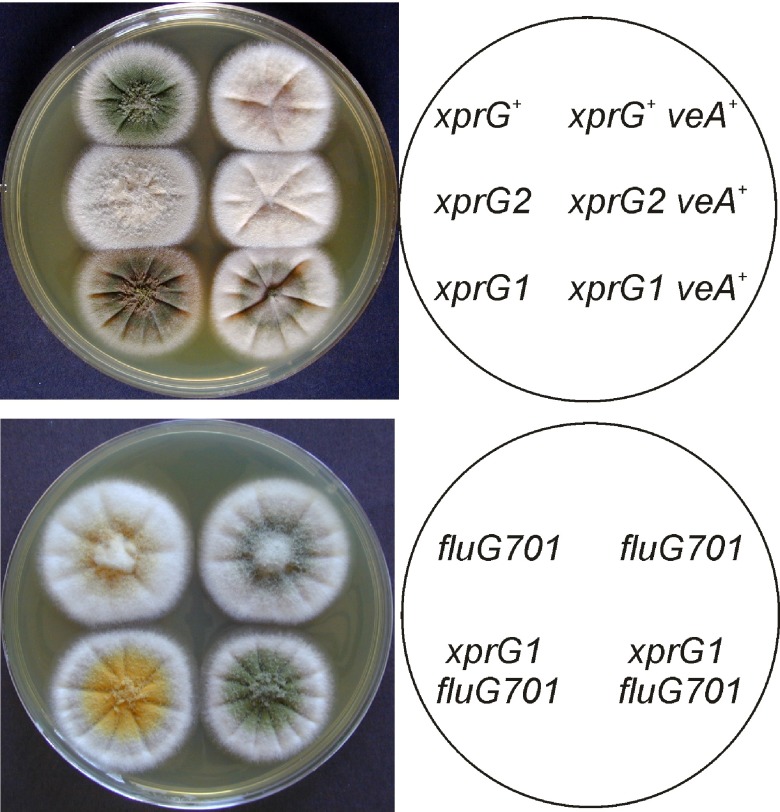
Interactions between the *xprG*, *veA* and *fluG* genes. **A.** Conidiation is suppressed by VeA in the dark but XprG1 partially restores conidiation in a *veA ^+^* strain. The plate was photographed after 3 days of growth on complete medium at 37°C. Light was excluded by wrapping the plate in aluminum foil. The full genotypes of the *xprG ^+^* (MH2), *xprG2* (MK198), *xprG1* (MK85), *xprG ^+^ veA ^+^* (WIM-126), *xprG2 veA ^+^* (MK565), and *xprG1 veA ^+^* (MK563) strains are given in Table 1. **B.** The *fluG* gene is involved in producing an extracellular signal for the induction of conidiophore development ^67^. The *fluG701* mutation is partially suppressed by the *xprG1* gain-of-function mutation. The full genotypes of the strains (top left MK593, top right MK592, bottom left MK595, bottom right MK594) are given in Table 1.

We investigated the genetic interactions between the *xprG* mutations and mutations in genes encoding key regulators of conidiophore development. VeA is a component of the light sensor which regulates the switch from sexual to asexual development. Laboratory strains of *A. nidulans* produce abundant asexual spores (conidia) in the absence of light because of a point mutation in the *veA* gene ^43^. To investigate the interaction between the *xprG* and *veA* genes, strains carrying the *xprG1* and *xprG2* mutations were crossed to a *ve ^+^*strain, which requires light to trigger asexual spore formation. When *xprG2 ve ^+^* segregants were grown in complete darkness, the colonies produced even fewer conidia than *xprG ^+^* *veA+* strains, whereas the *xprG1* gain-of-function mutation partially suppressed VeA-mediated repression of conidiophore development ( Figure 5). Programmed initiation of conidiation in surface cultures depends on FluG, but *fluG ^-^* mutants can be induced to undergo conidiophore development by nutrient stress ^52^. We found that the *xprG1* mutation partially suppresses the conidiophore development defect in the *fluG701* mutants ( Figure 5). In contrast, the *xprG1* mutation did not suppress the *brlA1* defect in conidiation.

Conidiophore length data To measure conidiophore length, strains inoculated into 1 cm^2^ blocks of complete medium on microscope slides as described by Larone (1995). Conidiophores were photographed at 400 x magnification after growth at 37^o^C on microscope slides. Measurements were carried out using the ImageJ program (http://rsbweb.nih.gov/ij/).

Conidial number data The number of asexual spores (conidia) per mm^2^ produced by A. nidulans strains MH2 (xprG^+^), MK422 (xprGΔ) and MK85 (xprG1) was determined by removing three plugs from colonies on complete medium containing 2.2% agar. The conidia from each plug were suspended in a solution of 0.01% TWEEN80 and counted in a haemocytometer. The experiment was repeated a total of four times using different batches of medium.

### Glucose uptake

**Figure 6.**
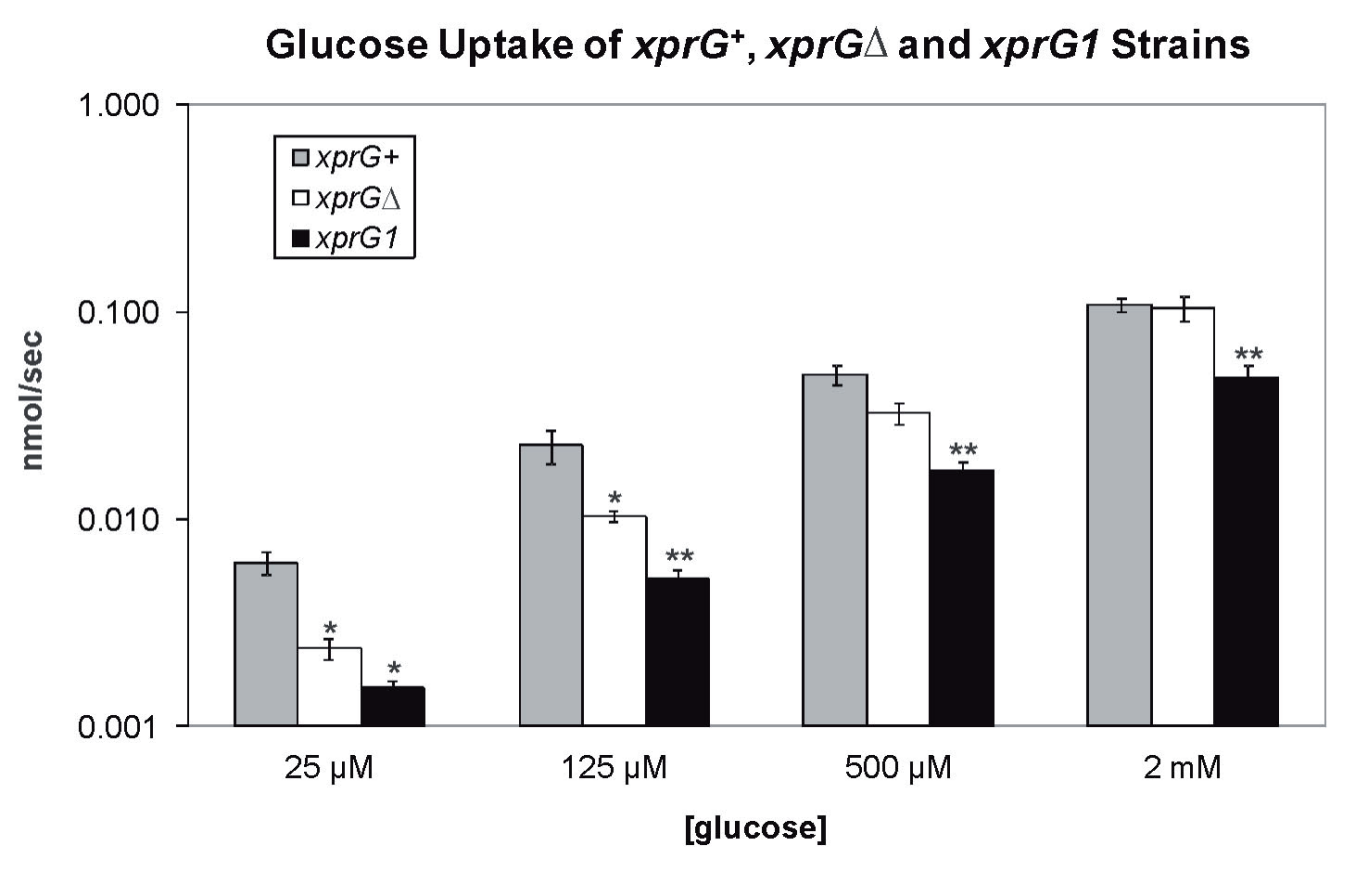
Glucose uptake, in 2.5 × 10 ^7^ germinating conidia, in the first 60 s after transfer to 25 µM, 125 µM, 500 µM or 2 mM glucose. The results are the average for four ( *xprGΔ1, xprG1*) and five ( *xprG ^+^*) experiments and standard errors are shown. The rate of glucose uptake was compared with the uptake of the *xprG ^+^* strain at each concentration of glucose using an unpaired t-test. Values which differed significantly from the value for the *xprG ^+^* strain are indicated with asterisks (*p < 0.5, **p < 0.1) The full genotypes of the *xprG ^+^* (MH2), *xprGΔ* (MK422) and *xprG1* (MK85) strains are given in Table 1.

The *Aspergillus niger mstA* gene encodes a high-affinity sugar transporter that is highly expressed during carbon starvation and repressed by glucose ^53^. The *A. nidulans* homologue of *mstA* was among the top five genes that were up-regulated in response to carbon starvation in an *xprG ^+^* strain in Experiment 1, and was down-regulated in the *xprGΔ1* mutant. The effect of *xprG* loss- and gain-of-function mutations on glucose transport was examined ( Figure 6). In the *xprGΔ1* mutant, glucose uptake was significantly reduced when low levels of glucose were present but was unaltered when the concentration of glucose was high, indicating that only high-affinity glucose uptake was decreased. Both high- and low-affinity uptake of glucose was reduced in the *xprG1* gain-of-function mutant.

Glucose uptake raw data The uptake of D-[U-^14^C] glucose (10.6 GBq/mmol, Amersham) was measured in germinating conidia of A. nidulans strains MH2 (xprG^+^), MK422 (xprGΔ) and MK85 (xprG1) as described previously by McCabe et al. (2003). Glucose uptake was measured in aliquots of 2.5 x 10^7^ germinating conidia after transfer to media containing 0.025, 0.125, 0.5 or 2 mM glucose.

### Autolysis

**Figure 7.**
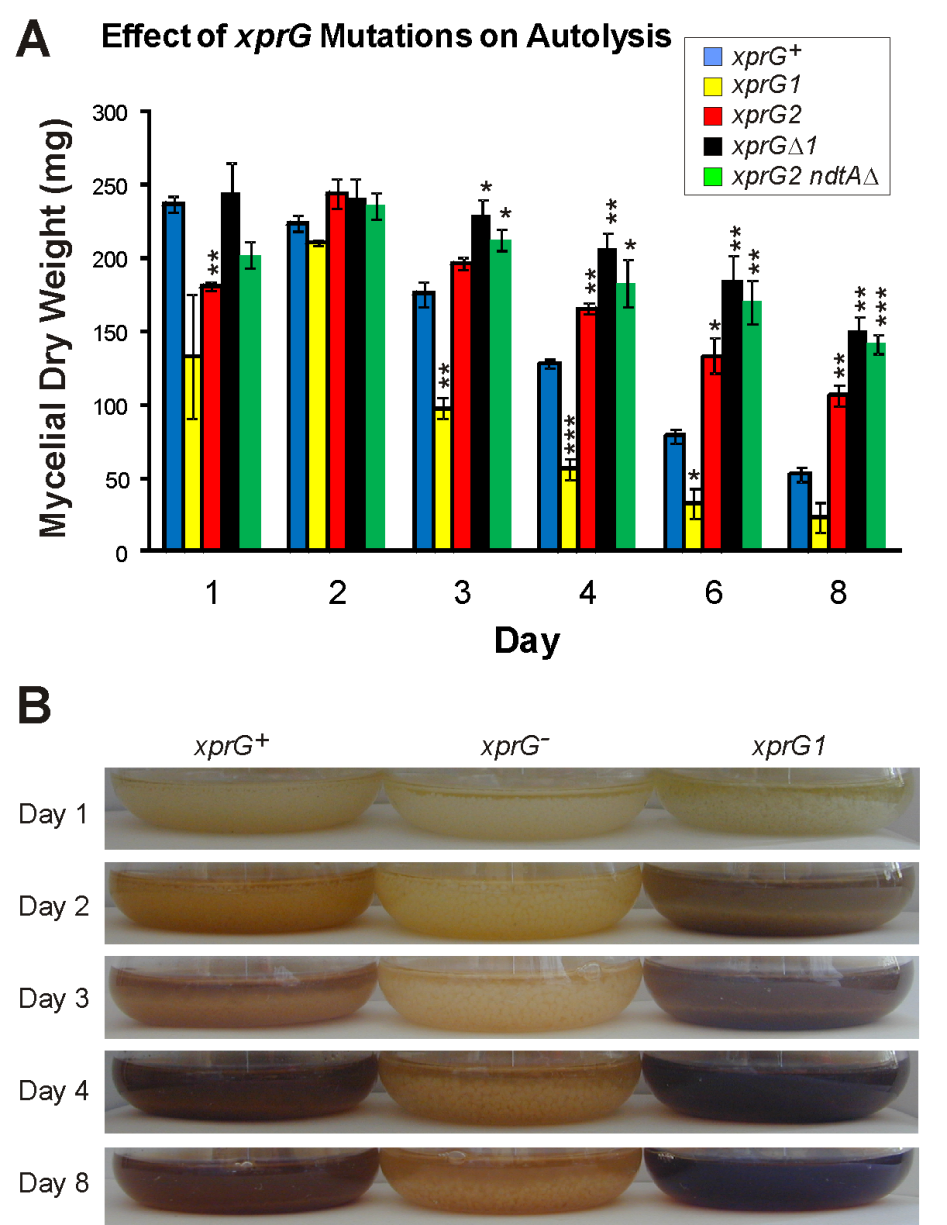
Effect of the *xprG2/xprGΔ1* loss-of-function and *xprG1* gain-of-function mutations on autolysis. Loss of mycelial mass ( **A**) and changes in the appearance of cultures ( **B**) were monitored for 8 days in submerged cultures inoculated with the same number of conidia. The results in A are the average for the three experiments and standard errors are shown. The mycelial mass at each time point was compared with the mass of the *xprG ^+^* strain using an unpaired t-test. Values which differed significantly from the value for the *xprG ^+^* strain are indicated with asterisks (*p < 0.5, **p < 0.1, ***p < 0.001) The full genotypes of the *xprG ^+^* (MH2), *xprG1* (MK85), *xprG2* (MK198), *xprGΔ1* (MK422) and *xprG2 ndtAΔ* (MK505) strains are given in Table 1.

The *chiB* gene, which plays an important role in autolysis, was among the top five genes that were up-regulated in response to carbon starvation in the *xprG ^+^* strain in Experiment 1, and was down-regulated in the *xprGΔ1* mutant. Production of extracellular proteases also increases during autolysis ^54^. The genes encoding two extracellular proteases, PrtA and PepJ, were down-regulated in the *xprGΔ1* mutant. Cultures of the *xprG1* and *xprG2* mutants were observed over a period of eight days to determine whether XprG plays a role in autolysis, which occurs in stationary, submerged cultures of *A. nidulans* after carbon source depletion ^45^. The disintegration of mycelial pellets, decline in mycelial mass, increase in culture medium turbidity due to hyphal fragmentation and accumulation of brown pigment which accompany autolysis occurred more rapidly in the *xprG1* gain-of-function mutant. In contrast, mycelial pellets were still present in the cultures of the *xprG2* and *xprGΔ1* mutants (the two *xprG ^-^*genotypes) after 8 days and there was no evidence of hyphal fragmentation or pigment accumulation ( Figure 7). These results indicate that XprG is required for autolysis in response to carbon starvation. Thus, XprG, like Vib-1 of *N. crassa* has a role in regulating programmed cell death.

The microarray experiments showed that expression of the *hpdA* gene was reduced in the *xprGΔ1* mutant. The *A. fumigatus hppD* gene is the ortholog of the *A. nidulans hpdA* gene and has been shown to be essential for the production of pyomelanin ^49^. A *ΔhppD* mutant has colourless mycelia and does not release pyomelanin in liquid mediuam. Thus, it is likely that the pale mycelia and absence of released pigment in the *xprG* ^-^mutants during autolysis is due to reduced *hpdA* expression.

Autolysis data To monitor autolysis, six flasks containing 50 mL of minimal medium, 10 mM ammonium tartrate and vitamin supplements were each inoculated with 3 x 10^8^ conidia and placed on an orbital shaker. Flasks were removed at 24 or 48 h intervals, the submerged mycelia harvested using Miracloth (Calbiochem/Merck). The dry weights of the mycelia are recorded in this file.

### Role of other Ndt80-like proteins in filamentous fungi

Ndt80 is a transcriptional activator required for progression through meiosis in *S. cerevisiae* ^9, 10^ whereas *A. nidulans* mutants lacking a functional copy of the *xprG* gene are able to complete meiosis. *S. cerevisiae* is unusual among ascomycete fungi in that it possesses only one transcription factor in this class ( Table 5). In *A. nidulans*, a second putative member of this class (AN6015) shows greater similarity to Ndt80 (17.1% identity overall and 23.5% in the DNA-binding domain) than does XprG (12.4% identity overall and 13.8% identity in the DNA-binding domain). To investigate the role of AN6015, the gene was disrupted. Strains carrying a disrupted copy of AN6015 could be crossed to wild-type strains but no cleistothecia (fruiting bodies) were observed when AN6015Δ mutants were crossed. These results suggest that AN6015 is required for sexual reproduction in *A. nidulans* and, as in *S. cerevisiae*, mutations in AN6015 are recessive. We suggest that AN6015 be named NdtA.

**Table 5.** The Ndt80 class of p53-like transcriptional activators in fungi.

Phylum	No. of genes encoding Ndt80-like proteins ^*a*^	Species
Basidiomycota	0	*Cryptococcus neoformans*
	0	*Coprinus cinereus*
	0	*Phanerochaete chrysosporium*
	0	*Postia placenta*
	0	*Puccinia graminis*
	1	*Ustilago maydis*
Ascomycota	0	*Schizosaccharomyces pombe*
	1	*Saccharomyces cerevisiae*
	2	*Aspergillus nidulans*
	2	*Aspergillus flavus*
	2–3	*Candida albicans*
	3	*Aspergillus fumigatus*
	3	*Magnaporthe oryzae*
	3	*Neurospora crassa*
	3	*Fusarium graminearum*
	4	*Fusarium oxysporum*
Chytridiomycota	2	*Batrachochytrium dendrobatidis*
	2	*Spizellomyces punctatus*
Zygomycota	5	*Phycomyces blakeleeanus*
	6	*Mucor circinelloides*
	7	*Rhizopus oryzae*

^*a*^Genome sequences were obtained from the Fungal Genome Initiative of the Broad Institute with the exception of the *P. chrysosporium*, *P. placenta* and *P. blakeleeanus* sequences which were from the DOE Joint Genome Institute.

**Figure 8.**
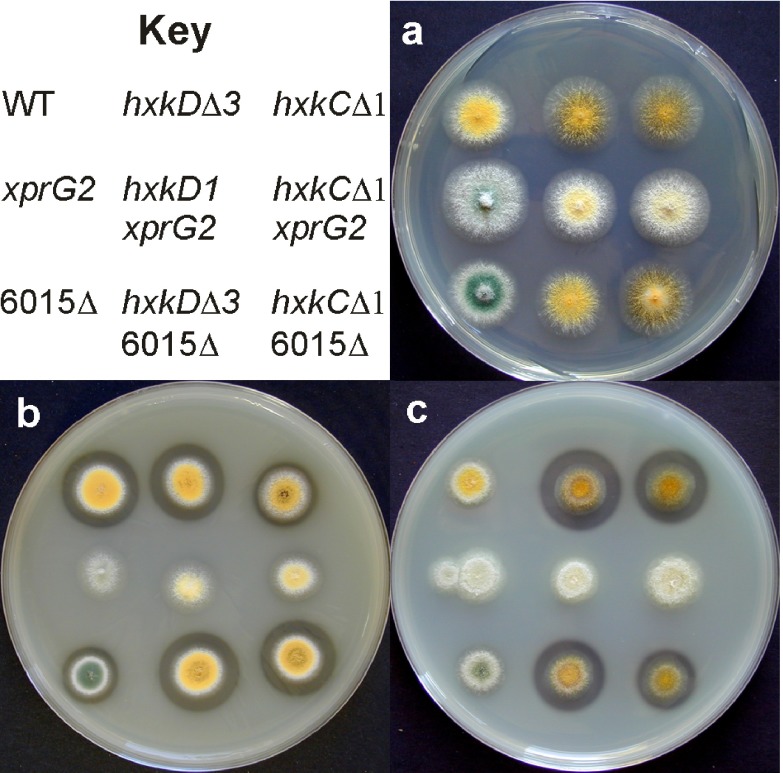
Phenotype of the *AN6015Δ* gene disruption mutant. Colony morphology and extracellular protease production of wild-type and mutant strains on ( **A**) minimal medium ( **B**) medium containing milk as a nitrogen source and ( **C**) medium containing milk as a carbon source. The clear halo surrounding colonies on medium containing milk is due to extracellular protease activity. The full genotypes of strains MH97 (WT), MK198 ( *xprG2*), MK481 (6015Δ), MK320 ( *hxkDΔ3*), MK186 ( *hxkD1* *xprG2*), MK532 ( *hxkDΔ3* 6015Δ), MK388 ( *hxkCΔ1*), MK408 ( *hxkCΔ1 xprG2*), and MK531 ( *hxkCΔ* 6015Δ) are given in Table 1.

Unlike *xprG* loss-of-function mutations, *ndtAΔ* does not affect conidial pigmentation ( Fig 8A), prevent extracellular protease production or suppress mutations in *hxkC* and *hxkD* ( Figure 8B and Figure 8C). If no ammonium is present, wild type strains produce a halo, due to extracellular protease activity, on medium containing milk as a nitrogen source. The *ndtAΔ* mutant also displays a halo but the *xprG2* mutant, which is protease-deficient, does not when grown on medium containing milk as a nitrogen source ( Figure 8B). Extracellular protease activity is low on medium containing milk as a carbon source, as carbon starvation is required to stimulate extracellular protease production when ammonium is present ^3^. The *hxkCΔ* and *hxkDΔ* mutants have elevated levels of extracellular protease and produce large halos on this medium ^1, 2^. The *xprG2* mutation suppresses this phenotype but the *ndtAΔ* mutation does not ( Figure 8C). *xprG2 ndtAΔ* double mutants had the same pale conidia as *xprG2* strains. Like the *xprG2* single mutant, the *xprG2 ndtAΔ* double mutant produced no halo on medium containing milk as a carbon or nitrogen source and did not undergo autolysis in response to nutrient stress ( Figure 7).

## Discussion

The transcriptional profiling data reported here reveal that XprG plays a major role in the activation of gene expression in response to carbon starvation. More than 37% of the 197 probes that hybridized to transcripts that were significantly up-regulated during carbon starvation, were down-regulated in the *xprGΔ1* mutant. This proportion is higher if less stringent criteria are used to identify differentially regulated transcripts; 60% of the transcripts up-regulated during carbon starvation show more than a two-fold decrease in transcript levels in the *xprGΔ1* mutant and 91% show at least some decrease. In contrast, less than 5% of the 319 probes that hybridized to transcripts that were down-regulated during carbon starvation were up-regulated in the *xprGΔ1* mutant and none were down-regulated. As XprG is a putative transcriptional activator, it is not surprising that it does not appear to be involved in repression of gene expression during carbon starvation. Secondary effects ( *e.g.* down-regulation of repressors) may be responsible for the few transcripts ^14^ that are down-regulated during carbon starvation and up-regulated in the *xprGΔ1* mutant. XprG also does not appear to play a role in regulating gene expression during growth in medium containing glucose as a carbon source.

HxkC and HxkD are hexokinase-like proteins which are negative regulators of extracellular protease production and may modulate the activity of XprG ^1, 3^. It has previously been reported that contrary to expectations, *hxkD* transcript levels increase during carbon starvation ^1^. The microarray data reported here showed that the *hxkC* gene, is also up-regulated during carbon starvation, and that increased expression of *hxkC* is dependent on XprG. It was not expected that *hxkC* and *hxkD* transcript levels would increase during carbon starvation, because HxkC and HxkD are negative regulators and production of extracellular proteases increases during carbon starvation. As noted previously, these results could indicate that HxkC and D have other functions during carbon starvation ^1^.

We have shown here that XprG regulates the expression of *brlA*, a key regulator of conidiophore development, in submerged cultures during carbon starvation. However, conidiophore development is essentially normal in *xprG ^-^* mutants grown on solid media and can be induced by carbon starvation in submerged cultures. Thus, the reduction of *brlA* expression observed in the *xprGΔ1* mutant is not sufficient to block conidiophore development. Nevertheless, the genetic evidence suggests that XprG plays some role in triggering asexual development as the *xprG1* mutation stimulates conidiophore development in a *veA ^+^*strain incubated in the dark and in a *fluG701* mutant.

Secondary metabolism and asexual/sexual development are linked in filamentous fungi. XprG appears to be a member of a group of regulatory proteins that control both secondary metabolism and development (reviewed in Bayram *et al.* ^55^). This group includes the light regulator VeA, which is required for sexual development ^43^ and has been shown to regulate sterigmatocystin production ^56^, LaeA, the global regulator of secondary metabolism ^57^ which is also required for asexual development ^58^, and components of a heterotrimeric G protein signaling pathway which is required for both asexual development and sterigmatocystin production ^59^. All of the proteins in this group act upstream of BrlA, the transcription factor that activates genes required for conidiophore development ^60^, but is not required for sterigmatocystin production ^61^. The *A. nidulans* homologue of *S. cerevisiae* Ime2 protein kinase is also a member of this group. An *imeBΔ* null mutant does not produce sterigmatocystin and overproduces sexual fruiting bodies in light in a *veA* ^+^ strain ^62^. In *S. cerevisiae* Ime2 activates transcription of Ndt80 and also controls Ndt80 activity through phosphorylation ^63^. XprG, as an Ndt80-like protein, could be a target of ImeB in *A. nidulans*.

In addition to the link between asexual development and secondary metabolism in *A. nidulans*, there is a link between asexual development and autolysis ^46, 54, 64^. Thus, XprG may play a direct role in regulating autolysis through regulation of chitinase (ChiB), extracellular proteases (PrtA, PepJ) and other hydrolytic enzymes or XprG could act indirectly through BrlA, which is involved in the induction of autolysis ^54^.

The *xprG1* gain-of-function mutant had previously been shown to have the reverse phenotype to *xprG ^-^* mutants with respect to extracellular protease and pigment production ^7^. Here we show that the *xprG1* mutation leads to accelerated autolysis and increased penicillin production, whereas autolysis and penicillin production is reduced or absent in an *xprG ^-^* mutant. Likewise, conidiation is increased in an *xprG1 veA ^+^* strain but decreased in an *xprG ^-^* *veA ^+^* strain. In contrast, glucose uptake and sterigmatocystin levels were reduced in both the *xprG1* and *xprG ^-^* mutants. The reason for this difference in phenotypic effect is not known. The *xprG1* allele contains a missense mutation (R186W) in the putative DNA-binding domain of XprG ^7^. It may be that this amino acid substitution increases the affinity of the XprG1 for some binding sites but decreases the affinity for others. Missense mutations with this type of gene specificity effect have been documented in the DNA-binding domain of AreA, the *A. nidulans* regulator of genes involved in nitrogen metabolism ^65^.

We have shown that the two genes encoding Ndt80-like proteins in *A. nidulans* perform different functions. Among fungi, there is considerable variation in the number of genes in the *NDT80* family ( Table 5). Most basidiomycetes and the unicellular ascomycete *Schizosaccharomyces pombe* do not possess any genes encoding Ndt80/PhoG-like proteins. In contrast, the zygomycetes have large numbers of these genes. The number of *NDT80*-like genes varies within genera (e.g. *Aspergillus*) and even within the same species (e.g. *Candida albicans*). As most ascomycetes have a gene similar to *NDT80* and one or more genes similar to *xprG* (data sourced from the Fungal Genome Initiative), it seems likely that the unicellular *S. cerevisiae* has lost the *xprG*-like gene.

The p53-like transcription factor superfamily ( http://supfam.org/) is comprised of seven families containing the following DNA-binding domains: p53, Rel/Dorsal, T-box, STAT, Runt, Ndt80, and the LAG-1/CSL. Many of the proteins in this superfamily, including MRF (myelin gene regulatory factor), a mammalian member of the Ndt80 family, are involved in development. The Ndt80 and LAG-1 families include both animal and fungal proteins and the Ndt80 family is also found in the slime molds *Dictyostelium discoideum* and *Dictyostelium purpureum*. The Ndt80 family is present in all ascomycete fungi, with the exception of the *Schizosaccharomyces* species, but is absent from most of the basidiomycete fungi that have been sequenced to date. In contrast, LAG-1 family members are found in all basidiomycetes but are lacking in all ascomycetes except *Schizosaccharomyces* species.

We have previously proposed that the common feature of fungal p53-like proteins is a role in nutrient sensing, and this may be the original role for this group of transcriptional activators ^7^. It has recently been shown that Ndt80 is involved in resetting lifespan during meiosis and transient expression of *NDT80* extends the lifespan of aging yeast cells ^11^. Pathways responsible for the response to nutrient status appear to play an important role in controlling lifespan ^66^. We speculate that the ability of Ndt80 to sense nutrient status could be crucial in determining lifespan.

## Figures and Tables

**Supplementary Table 1.** Oligonucleotides used in qRT-PCR experiments.

Gene	Oligonucleotide sequence ^a^	Position ^b^
*actA*	5´-AGAGGAAGTTGCTGCTCTCG-3´ (F)	6
	5´-GGATACCACGCTTGGACTGT-3´ (R)	193
*benA*	5´-CGTGAGATCGTTCACCTTCA-3´ (F)	4
	5´-GAAGGTCGGAGGTACCATTG-3´ (R)	127
*brlA*	5´-TCATCAAGCAGGTGCAGTTC-3´ (F)	941
	5´-CGTATAGTGGGCGTTGAGGT-3´ (R)	1116
*chiB*	5´-ACGATCAGCAGGCTCAGAAC-3´ (F)	425
	5´-TCTCCTGTAGCCGGAGCTTA-3´ (R)	568
*ppgA*	5´-TGCCGCTGAATTACAACATC-3´ (F)	66
	5´-CGGAACCTGCACCATCTATT-3´ (R)	212
*tdiB*	5´-GATGGACCTGATTGCTTCGT-3´ (F)	606
	5´-TGTGCAGGTAGCATTTGACC-3´ (R)	727
*veA*	5´-GAGCTTGTGGTCAAGGTTCG-3´ (F)	23
	5´-GACGCTCGGGTTCTAGAGTG-3´ (R)	196

^a^The sequences labeled (R) are complementary to the sense strand.

^b^The position of the 5´ end of each primer in the coding region of the gene (excluding introns) is given. The sequences were obtained from the *Aspergillus* Genome Database.
