# Supplementary material for: A p53-like transcription factor similar to Ndt80 controls the response to nutrient stress in the filamentous fungus, Aspergillus nidulans
Source: F1000Res. 2013 Mar 4;2:72. [Version 1] doi: 10.12688/f1000research.2-72.v1 (PMC3821154; doi:10.12688/f1000research.2-72.v1)
Supplement: Peer review history

# Peer review: Referee response for version 1

**Published:** 2013-04-08

**Authors:** Michael Hynes

**Affiliations:** 1 Faculty of Science, Department of Genetics, The University of Melbourne, Melbourne, Australia

Carbon starvation is likely to be a common stress that fungi encounter in the environment. This group has previously identified the *xprG* gene, which contains a p53 like Ndt80 DNA binding domain, as being involved in the response to starvation. Here they have studied by microarrays the effects of *xprG* on the response to 16 hours of carbon starvation. They have verified some of the responses by qRT-PCR as well as physiological studies. 

Effects on glucose uptake, conidial and hyphal pigmentation, secondary metabolite production and autolysis were verified and are consistent with previous studies. The effects of an *xprG* gain of function mutation support the results. This work therefore provides strong support for XprG playing an important role in the response to starvation – a novel and significant result which adds to the large body of data relating to genes involved in development and secondary metabolite production in *A. nidulans*.

A further significant result is the finding that a second Ndt80 domain containing gene, AN6015- designated *ndtA*, when deleted results in loss of sexual development. This may be related to the known role of Ndt80 in meiosis in Saccharomyces cerevisiae. Interestingly an Ndt80 homolog has been found to be involved in biofilm formation in *Candida albicans* ( Cell 148, 126–138).

I have read this submission. I believe that I have an appropriate level of expertise to confirm that it is of an acceptable scientific standard.

# Peer review: Referee response for version 1

**Published:** 2013-03-25

**Authors:** Amir Sharon

**Affiliations:** 1 Department of Molecular Biology and Ecology of Plants, Tel Aviv University, Tel Aviv, Israel

The title and abstract are appropriate for the paper. The work presented in this paper is well planned, experiments are well designed and executed, and the analyses are comprehensive and provide clear answers to the main questions. Particularly, the interpretation of the micro array data, analysis of differentially expressed genes, and reference to most significantly changed gene/gene clusters between the wild type and mutant are excellent. 

The conclusions are all well supported by the data and interpreted in a conserved manner. The results are novel and interesting. All experiments are detailed and clear.

I have read this submission. I believe that I have an appropriate level of expertise to confirm that it is of an acceptable scientific standard.
